# MicroRNA 630 Represses NANOG Expression through Transcriptional and Post-Transcriptional Regulation in Human Embryonal Carcinoma Cells

**DOI:** 10.3390/ijms23010046

**Published:** 2021-12-21

**Authors:** Wing-Keung Chu, Li-Man Hung, Chun-Wei Hou, Jan-Kan Chen

**Affiliations:** 1 Healthy Aging Research Center, Chang Gung University, Taoyuan 333, Taiwan; d9701303@cgu.edu.tw (W.-K.C.); lisahung@mail.cgu.edu.tw (L.-M.H.); romeomonkey@msn.com (C.-W.H.); 2 Department and Graduate Institute of Biomedical Sciences, College of Medicine, Chang Gung University, Taoyuan 333, Taiwan; 3 Kidney Research Center, Chang Gung Memorial Hospital, Linkou 333, Taiwan; 4 Department of Physiology, College of Medicine, Chang Gung University, Taoyuan 333, Taiwan

**Keywords:** embryonal carcinoma cells, MicroRNA, NANOG, 3′UTR

## Abstract

The pluripotent transcription factor NANOG is essential for maintaining embryonic stem cells and driving tumorigenesis. We previously showed that PKC activity is involved in the regulation of NANOG expression. To explore the possible involvement of microRNAs in regulating the expression of key pluripotency factors, we performed a genome-wide analysis of microRNA expression in the embryonal carcinoma cell line NT2/D1 in the presence of the PKC activator, PMA. We found that MIR630 was significantly upregulated in PMA-treated cells. Experimentally, we showed that transfection of MIR630 mimic into embryonal carcinoma cell lines directly targeted the 3′UTR of OCT4, SOX2, and NANOG and markedly suppressed their expression. RNAhybrid and RNA22 algorithms were used to predict miRNA target sites in the *NANOG* 3′UTR, four possible target sites of MIR630 were identified. To examine the functional interaction between MIR630 and *NANOG* mRNA, the predicted MIR630 target sites in the *NANOG* 3′UTR were deleted and the activity of the reporters were compared. After targeted mutation of the predicted MIR630 target sites, the MIR630 mimic inhibited NANOG significantly less than the wild-type reporters. It is worth noting that mutation of a single putative binding site in the 3′UTR of *NANOG* did not completely abolish MIR630-mediated suppression, suggesting that MIR630 in the *NANOG* 3′UTR may have multiple binding sites and act together to maximally repress NANOG expression. Interestingly, MIR630 mimics significantly downregulated *NANOG* gene transcription. Exogenous expression of OCT4, SOX2, and *NANOG* lacking the 3′UTR almost completely rescued the reduced transcriptional activity of MIR630. MIR630 mediated the expression of differentiation markers in NT2/D1 cells, suggesting that MIR630 leads to the differentiation of NT2/D1 cell. Our findings show that MIR630 represses NANOG through transcriptional and post-transcriptional regulation, suggesting a direct link between core pluripotency factors and MIR630.

## 1. Introduction

A number of transcription factors have been shown to play critical roles in the self-renewal and maintenance of pluripotency in embryonic stem cell (ESCs), among them OCT4, NANOG, and SOX2 [1] have been shown to be essential. Cooperativity between OCT4 and SOX2 has been shown to drive the expression of pluripotent genes. For instance, transcription of *NANOG* has been shown to be regulated by the OCT4/SOX2 complexes through a highly conserved adjacent pair of OCT4 and SOX2 binding elements in the *NANOG* promoter [2]. NANOG is selectively expressed in undifferentiated pluripotent cells, which activates the repressors and suppresses the activators of differentiation in a precisely controlled manner [3]. Expression of NANOG is involved in the pathways that form a reciprocal regulatory circuit to balance the self-renewal and differentiation of ESCs, which is critical for development, tissue homeostasis, and tumorigenesis [4,5]. Cancer cells and ESCs have been shown to share many key biological properties, including high proliferative potential, which is essential for embryogenesis and tumor development. NANOG and its pseudogene, NANOGP8, are expressed not only in human germ cell tumors [6] but also in several human cancers, including colorectal cancer [5], seminoma and breast carcinoma [7], oral cavity carcinoma [8], ovary carcinoma [9], renal carcinoma [10], and malignant cervical epithelial cells [11]. Overexpression of NANOG in cancer is frequently associated with advanced stage, lymph node metastasis, poor differentiation, and resistance to treatment and has been shown to be strongly correlated with poor prognosis [5,12]. Nevertheless, little is known about how the expression of NANOG is regulated. We previously reported that Protein Kinase C (PKC) activity is involved in the regulation of NANOG expression [13]. We showed that by inhibiting PKC activity, NANOG expression was upregulated in six human cancer cell lines. Knockdown experiments showed that PKCα and σ are the two isozymes that exert most of the effects. Moreover, in constitutively NANOG-overexpressing human embryonal carcinoma cells (NT2/D1 and NCCIT), NANOG expression was repressed by PKC activation. Ectopic PKCα expression in NT2/D1 cells suppressed the expression of NANOG and TRA-1-60, a human pluripotent stem cell marker. NANOG expression is of cellular significance, as knockdown of endogenous NANOG expression in NT2/D1 cells inhibits cell proliferation. Since NANOG expression appears to play a regulatory role in tumor growth, we suggest that the PKC-NANOG pathway may be involved in tumor development and progression.

MicroRNAs (miRNAs) are a large family of non-coding RNAs, typically 19–25 nucleotides in length. They regulate gene expression at the post-transcriptional level and are one of the crucial regulators necessary to maintain ESC self-renewal. Genetic deletion of the major miRNA processing enzymes DGCR8 [14] or Dicer [15,16] in murine ESCs results in loss of their pluripotency and they remain differentiation defective. Therefore, miRNAs and their underlying regulatory mechanisms are of great importance for the self-renewal and pluripotency of ESCs.

In the current study, we investigated the possible involvement of miRNAs in the regulation of NANOG expression by PKC. We first identified 20 miRNAs that were significantly upregulated in PKC activator-treated NT2/D1 cells. Computational predictions indicated that miRNA 630 (MIR630) targeted multiple core pluripotency factors, including NANOG, OCT4. and SOX2. Further experimental work showed that MIR630 directly targeted the 3′UTR of *NANOG* and repressed its expression. This regulation was found to be at the transcriptional and post-transcriptional levels.

## 2. Results

### 2.1. Activation of PKC Upregulates MIR630 Expression

Recent studies have shown that miRNAs play a critical role in the regulatory circuit that controls pluripotency and self-renewal of ESCs [17,18]. During ES cell differentiation, miRNA expression patterns were found to be altered, which provides valuable clues to their possible involvement in regulating pluripotency of ESCs [19]. In this study, we explored the possible involvement of miRNAs in PKC activation-induced downregulation of NANOG in NT2/D1 cells.

We performed a genome-wide analysis of miRNA expression in NT2/D1 cells in the presence of PKC activator, phorbol 12-myristate 13-acetate (PMA), in an attempt to link this change to altered NANOG expression. MiRNA microarray was used to identify the miRNA species that were up- or down-regulated after PMA treatment (Table 1).

Among the miRNAs upregulated by PMA treatment, the expression of MIR630 was upregulated 28-fold, while MIR335-3p and MIR335-5p were upregulated by 17.1- and 21.9-fold, respectively, compared to untreated cells. The upregulation of MIR335-5p during mouse ESC differentiation is thought to play an important role in antagonizing OCT4-promoted mouse ESC self-renewal [20]. In addition, MIR29b-1*, a member of the MIR29 family predicted to target Krüppel-like factor 4 (KLF4) [21], a transcription factor required for the reprogramming of somatic cells into induced pluripotent stem cells, was also shown to be upregulated in PMA-treated NT2/D1 cells (7.4 fold).

### 2.2. MIR630 Is a Strong Candidate Repressor of Pluripotency Genes

The computational programs RNAhybrid [22] and RNA22 v1.0 [23] were used to predict miRNA target sites in the 3′UTRs of the *NANOG/POU5F1*(OCT4)/*SOX2*. One possible target site was identified in *POU5F1* and *SOX2*, while four target sites of MIR630 were identified in *NANOG* (Table 2). By expression analysis experiments, we found that the expression level of MIR630 was relatively low in NT2/D1 cells and significantly upregulated in PMA-treated NT2/D1 cells (Table 1). The effect of PKC activation on MIR630 expression was further confirmed in NT2/D1 cells. Bisindolylmaleimide I (BIM-I), a PKC inhibitor with high selectivity for PKCα, β1, β2, γ, δ, and ε isozymes, was used to demonstrate that the upregulation of MIR630 was specifically mediated by PKC activation. Cells were exposed to 50 nM PMA and pretreated with or without 2.5 µM of the PKC inhibitor BIM-I for 24 h. Total RNA was harvested and subjected to RT-qPCR analysis. As shown in Supplementary Figure S1, treatment of cells with 50 nM PMA enhanced the expression of MIR630 up to 3.6-fold. The upregulation of PMA was reversed after pretreatment of cells with the PKC inhibitor BIM-I, suggesting that activation of PKC promoted the expression of MIR630. Taken together, we suggest that MIR630 may be an important regulator of NANOG expression and, thus, cellular pluripotency.

### 2.3. Ectopic MIR630 Targets NANOG, POU5F1 (OCT4), and SOX2 3′UTR Reporters

**Figure 1:**
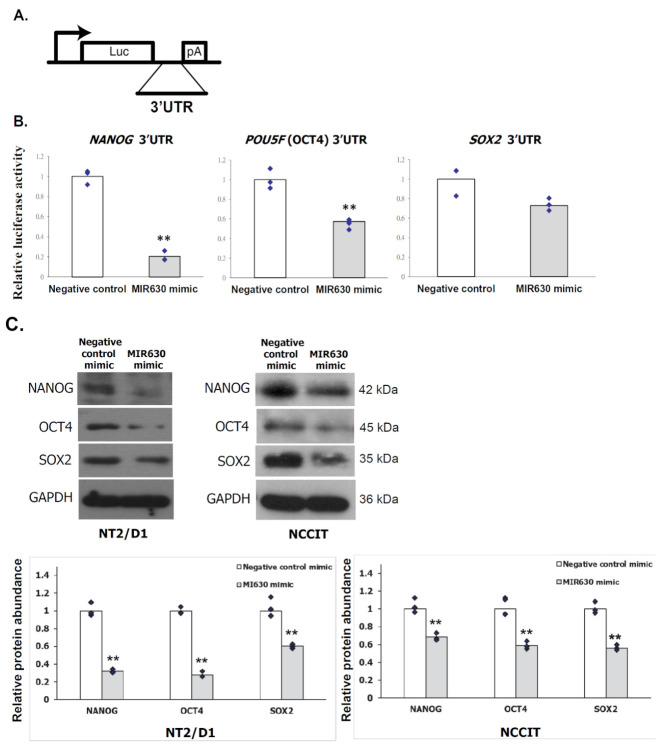
MIR630 specifically represses the reporter activity of its targets in the luciferase assay in embryonal carcinoma cells. (**A**). Structure of the 3′UTR reporters. Luc, firefly luciferase; pA, polyadenylation signal. (**B**). NT2/D1 cells were co-transfected with 30 nM MIR630 mimic and a luciferase reporter containing the 3′UTR. Two-tailed Student’s t-test was used for comparisons, where ** denotes *p* < 0.01. ♦ represent data points in Figure 1. (**C**). NT2/D1 cells and NCCIT cells were transfected with negative control miRNA or MIR630 mimic for 24 h. Total cell lysate was analyzed by Western blotting with antibodies against GAPDH, NANOG, OCT4, or SOX2. GAPDH was used to indicate that each lane was loaded with an equal amount of protein. Double asterisk indicates a significant difference between negative control mimic-transfected cells and MIR630 mimic-transfected cells. (*p* < 0.01, *t*-test).

To investigate whether NANOG, OCT4, and SOX2 are indeed targeted by MIR630, we constructed a luciferase reporter containing the 3′UTRs of these genes (Figure 1A). The luciferase reporter was cotransfected with mirVana miRNA mimic into NT2/D1 cells. A negative control with no homology to the human genome was used to monitor possible nonspecific expression effects. Compared to the negative control miRNA mimic, miRNA mimic of MIR630 reduced luciferase activity of wild-type *NANOG*, *POU5F1* (OCT4), and *SOX2* reporters ranging from 27% to 80% (Figure 1B). The results indicated that MIR630 significantly targets 3′UTRs of *NANOG* and *POU5F1*(OCT4) to regulate their expression.

A negative control mimic or miRNA mimic was transfected into human embryonal carcinoma cell lines, NCCIT and NT2/D1. Protein expression was examined by Western blotting. In NT2/D1 cells, the expression of NANOG, OCT4, and SOX2 proteins was inhibited by 69%, 70%, and 32%, respectively, after transfection with MIR630 mimic compared to control cells (Figure 1C). Similarly, MIR630 mimic inhibited the expression of NANOG, OCT4, and SOX2 proteins in NCCIT cells by 32%, 41%, and 44%, respectively, compared to control cells (Figure 1C). The results revealed that MIR630 mediated the post-transcriptional repression of NANOG, OCT4, and SOX2.

### 2.4. MIR630 Targets the Putative Binding Sites in the 3′UTR of NANOG

**Figure 2:**
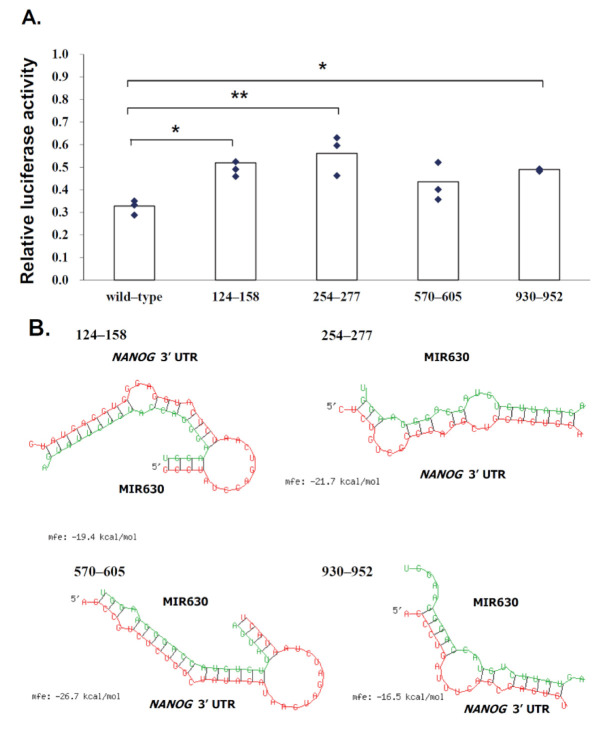
Mutations in the predicted MIR630 target sites exert significantly lower repression than wild-type reporter. (**A**). NT2/D1 cells were co-transfected for 24 h with negative control miRNA or MIR630 mimic and luciferase reporter containing wild-type and mutated *NANOG* 3′UTR. Numbers 124–158, 254–277, 570–605, and 930–952 represent *NANOG* 3′UTR positions. Cells were also cotransfected with pSV-β-galactosidase vector, and the β-galactosidase activity was used to normalize the luciferase activity. The relative luciferase activity is shown for reporter constructs containing wild-type 3′UTR or mutated 3′UTR in cells transfected with negative control miRNA or MIR630 mimic. Values were compared using one-way ANOVA and a post hoc test with Holm–Sidak, where * denotes *p* < 0.05 and ** denotes *p* < 0.01. ♦ represents data points. (**B**). Schematic representation of base pairing between putative *NANOG* 3′UTR binding sites and MIR630. RNAhybrid model presents the most energetically stable complementary base pairing between MIR630 and *NANOG* 3′UTR mRNA.

To examine the functional interaction between MIR630 and *NANOG* mRNA, the predicted MIR630 target sites in the *NANOG* 3′UTR region were deleted and the activity of the reporter was compared in negative control mimic or cells transfected with MIR630 mimic. As expected, MIR630 repressed 70% of the activity of the reporter construct containing the full-length wild-type *NANOG* 3′UTR (Figure 2). Correspondingly, we found that mutations in the putative binding sites (124–158, 254–277, and 930–952) resulted in an attenuated repressive effect by MIR630 mimic, leading to 52%, 32%, and 49% reductions in luciferase reporter activity (124–158, *p* < 0.05; 254–277, *p* < 0.01; 930–952, *p* < 0.05). In contrast, no statistically significant attenuated repressive effect was observed in the reporter that was deleted in 570–605.

In general, most miRNA target sites have a strong base pairing at the 5′ seed site. This site is referred to as the canonical site and is the site of mRNA–miRNA interaction involving the pairing of the seed region (positions 2–7). Among the putative binding sites, we showed that MIR630 forms the most stable base pairing with 570–605 in the *NANOG* 3′UTR (Figure 2B), which contains the highest free energy hybridization value. However, the interaction sites predicted in this study did not appear to involve the canonical perfect base pairing with the seed sequence.

Notably, mutation of a single putative binding site did not completely abolish the MIR630-mediated inhibition of reporter activity. This suggests that MIR630 may have multiple binding sites in the *NANOG* 3′UTR, all of which may be involved in MIR630-mediated downregulation of NANOG in a coordinated manner.

### 2.5. MIR630 Inhibits NANOG Transcription via Direct Repression of OCT4, SOX2, and NANOG Expression in NT2/D1 Cells

**Figure 3:**
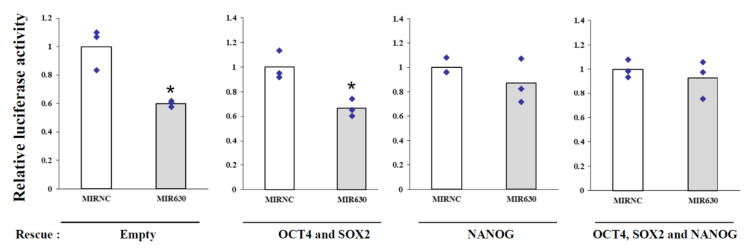
Ectopic OCT4, SOX2, and NANOG expression rescues MIR630-repressed NANOG transcriptional activity. Luciferase reporter containing the NANOG promoter region was cotransfected with a negative control mimic (MIRNC) or MIR630 plus OCT4, SOX2, or/and NANOG expression vectors for 24 h in NT2/D1 cells. Luciferase activity was normalized to β-galactosidase activity and indicated as the relative activity to the corresponding negative control mimic (assigned as value “1”). Comparisons were performed using a two-tailed Student’s t-test, where * denotes *p* < 0.05. ♦ represents data points.

The modulation of NANOG expression by MIR630 may be at the transcriptional and/or post-transcriptional level. Therefore, we employed reporter gene assays to first examine the transcriptional activity of the *NANOG* promoter. We cotransfected pGL3-NANOG-Luc [13] into NT2/D1 cells with negative control mimic or MIR630 mimic to examine the reporter activity, which contains a human *NANOG* promoter region from –993 to +231 relative to the *NANOG* transcriptional start site. Cotransfection of the pGL3-NANOG-Luc construct with the MIR630 mimic resulted in a significant 40% reduction in luciferase activity compared to cells transfected with negative control mimic (Figure 3).

*NANOG* transcription has also been reported to be regulated by the OCT4/SOX2 complexes through a highly conserved adjacent pair of composite Sox-Octamer element (−122 to −97 relative to the transcriptional start site) in the 5′-flanking region of the *NANOG* promoter [2]. We also previously reported that *NANOG* promoter activity is driven by a reduced cellular PKC activity, an effect that depends on the presence of the composite Sox-Octamer element [13]. In addition, NANOG autoregulates its expression by binding to its binding element in the proximal promoter (–81 to –63 relative to the transcriptional start site) [24].

To determine whether NANOG, OCT4, and SOX2 proteins are involved in *NANOG* transcription repressed by MIR630, we carried out “rescue” experiments in NT2/D1 cells. We assumed that NANOG, OCT4, and SOX2 are involved in MIR630-repressed *NANOG* transcription. Then, re-expression of NANOG, OCT4, and SOX2 could rescue the inhibitory effect of MIR630 on *NANOG* transcriptional activity. For this purpose, we used constructs containing pCMV-OCT4, pCMV-SOX2, and pCMV-NANOG expressing full-length cDNA but without the 3′UTR. Thus, this construct expressed ectopic OCT4, SOX2, and NANOG and should not be affected by the MIR630 putative binding sites. Cotransfection of MIR630 mimic and constructs overexpressing OCT4 and SOX2 in NT2/D1 cells partially restored the reduction in luciferase reporter activity induced by MIR630 transfection (Figure 3). Overexpression of NANOG did not completely abrogate the MIR630-mediated reduction in *NANOG* transcriptional activity. Consistently, transfection with ectopic OCT4, SOX2, and NANOG almost completely reversed the inhibitory effect of MIR630 on *NANOG* transcription in NT2/D1 cells. To confirm that pCMV constructs were not affected by MIR630, we transfected MIR630 mimic or negative control mimic into NCCIT cells together with PCMV constructs containing full-length *NANOG*, *POU5F1* (OCT4), and *SOX2* cDNAs. As shown in Supplementary Figure S2, the MIR630 mimics had no significant repressive effect on the expression of ectopic OCT4, SOX2, and NANOG. These results suggest that the repression of NANOG protein expression by MIR630 is at least partially regulated at the transcriptional level.

### 2.6. MIR630 Mediates the Expression of Differentiation Markers in NT2/D1 Cells

**Figure 4:**
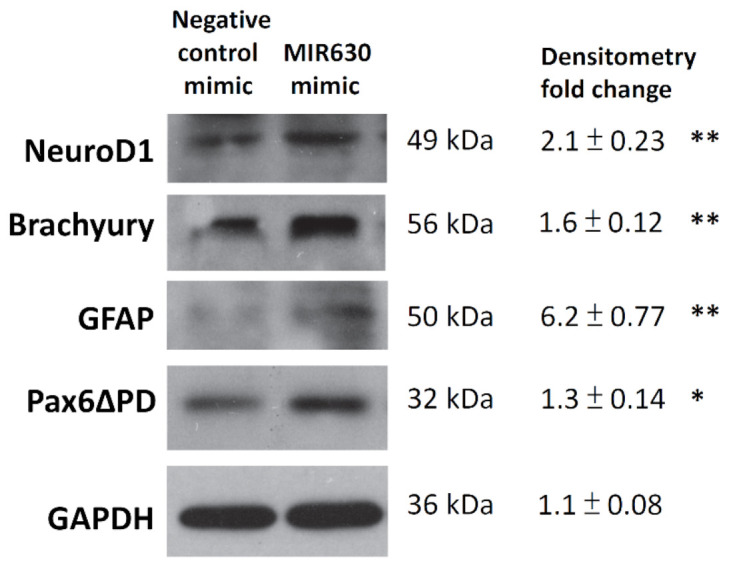
MIR630 mediates the expression of differentiation markers in NT2/D1 cells. NT2/D1 cells were stably transfected with either negative control miRNA mimic or MIR630 mimic for 3 days. Total cell lysates were prepared and analyzed by Western blotting with antibodies against NeuroD1, Brachyury, GFAP, Pax6, or GAPDH. To compare the relative band intensities, all immunoreactive bands were normalized to GAPDH by densitometry. The ratio in negative control miRNA-transfected cells was set to 1. Data are mean ± s.d. Two-tailed Student’s t-test was used, where * denotes *p* < 0.05 and ** denotes *p* < 0.01.

To further determine whether MIR630 plays a role in NT2/D1 cell differentiation, cells were transfected with negative control mimic or MIR630 mimic. After 3 days, total cellular proteins were extracted and the expression of some selected differentiation markers was analyzed by Western blot. NeuroD1, the earliest marker of neuronal differentiation, was found to be upregulated in MIR630-transfected cells (Figure 4). Moreover, brachyury, the earliest marker of mesodermal and endodermal differentiation was also increased. Glial fibrillary acidic protein (GFAP), a recognized marker of astrocyte differentiation, was also sharply induced. A 32/33k Da pax6 isoform, Pax6∆PD, was also upregulated in MIR630-transfected cells and this isoform was specifically detected in the developing eye [25]. These results suggest that MIR630 may lead to the differentiation of NT2/D1 cells.

## 3. Discussion

NANOG has been shown to be a central regulator in maintaining the self-renewal properties and pluripotency of human ESCs [17]. In addition to being expressed in ESCs, NANOG has been shown to be expressed in many types of human cancers [5,6,7,8,9,10,11]. Thus, the regulation of NANOG expression may also be important in tumorigenesis.

Accumulating evidence strongly suggests that alterations in miRNA expression are associated with cancer initiation and progression. In addition to regulating the expression of tumor suppressors and oncogenes, recent studies have indicated that miRNAs play a role in the maintenance of pluripotency and cellular reprogramming in ESC. On the other hand, NANOG, OCT4, and SOX2 have all been recognized to play a pivotal role in pluripotency maintenance. Therefore, it is important to investigate whether there is cross-talk between these two regulatory systems. In the present study, we scanned the genomic loci of *NANOG*, *POU5F1* (OCT4), and *SOX2* for putative target sites complementary to MIR630. We identified putative target sites of MIR630 in the 3′UTR of *NANOG*, *POU5F1* (OCT4), and *SOX2* mRNA. In this regard, we showed that PKC activators induce the expression of MIR630, while MIR630 mimic represses the luciferase activity of *NANOG*, *POU5F1,* and *SOX2* 3′UTRs reporters. We further showed that ectopic MIR630 mimic directly targets *NANOG*, *POU5F1,* and *SOX2* 3′UTRs to regulate their expression. Furthermore, after targeted mutation of the predicted MIR630 target sites in the 3′UTR of *NANOG*, the MIR630 mimic inhibited *NANOG* significantly less than the wild-type reporters. Our results suggest that MIR630 negatively regulates the expression of NANOG, OCT4, and SOX2 in a post-transcriptional manner and that their upstream regulator may be PKC.

**Figure 5:**
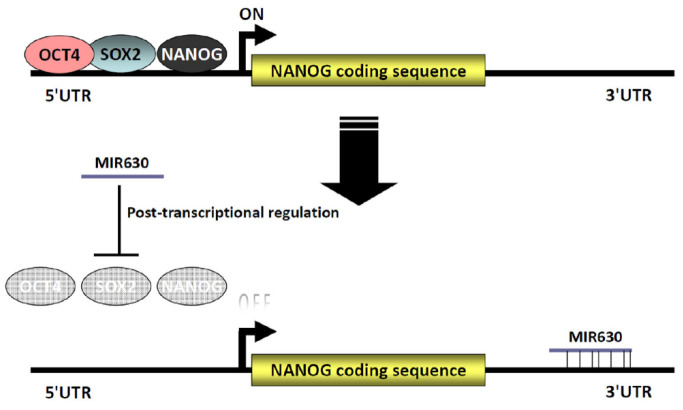
A schematic diagram depicting the MIR630-repressed NANOG expression via transcriptional and post-transcriptional regulation. We propose that MIR630 represses NANOG through transcriptional and post-transcriptional regulation. Transcription of *NANOG* is regulated by the OCT4/SOX2 complexes through an adjacent pair of highly conserved composite Sox-Octamer element in the 5′-flanking region of the *NANOG* promoter. In addition, NANOG autoregulates its expression by binding to the proximal promoter. In the present study, we showed that MIR630 directly targets the *NANOG*, *POU5F1,* and *SOX2* 3′UTRs to repress their expression. NANOG was further reduced due to the downregulation of NANOG, OCT4, and SOX2 levels.

In a previous study, we found that PKC inhibitors’ increased NANOG expression coincided with enhanced expression of OCT4 and SOX2. Furthermore, site-specific mutations in the composite Sox-Octamer element in the *NANOG* promoter vector abolished the upregulation of promoter activity by PKC inhibitors. Consistently, knockdown of OCT4 and SOX2 diminished the PKC inhibitor-induced enhancement of NANOG expression, suggesting that the OCT4/SOX2 complexes are involved in the downregulation of NANOG expression by PKC. Furthermore, NANOG was reported to autoregulate its expression by binding to its proximal downstream promoter [24]. The results showed that NANOG, OCT4, and SOX2 act as positive transcriptional regulators of NANOG expression through cooperative interactions. In the present study, MIR630 repressed the expression of NANOG, OCT4, and SOX2 in a post-transcriptional regulatory manner; therefore, we investigated the possible involvement of NANOG, OCT4, and SOX2 in *NANOG* transcription by MIR630. The results showed that MIR630 mimic significantly reduced the *NANOG* transcription compared to negative control mimic-transfected cells. Intriguingly, ectopic expression of *POU5F1*(OCT4), *SOX2*, and *NANOG* lacking the 3′UTR almost completely restored the reduced transcription of MIR630. These results strongly suggest that NANOG and OCT4–SOX2 complexes are involved in MIR630-repressed *NANOG* gene transcription. Taken together, this suggests that MIR630 represses NANOG expression at the transcriptional and post-transcriptional levels (Figure 5). Since high NANOG expression is associated with poor differentiation of solid tumors, it was interesting to know whether MIR630 promotes the differentiation of embryonal carcinoma cells. The results clearly showed that MIR630 induced the expression of differentiation markers in NT2/D1 cells, suggesting that MIR630 leads to the differentiation of NT2/D1 cells and may downgrade their pluripotent status (Figure 4.)

The cellular signals mediating the up-regulation of MIR630 expression are unclear. Since PKC is a well-known mediator of MAPK/AP-1 pathway stimulation [26,27], it is speculated that PKC-mediated MIR630 expression may function through MAPK/AP-1 activation. It has been reported that the expression of many MAPKs-regulated genes are dependent on NF-κB activity [28]. Therefore, NF-κB may also be involved in the role of PKC. In summary, the present study suggests that MIR630 negatively regulates NANOG expression through transcriptional and post-transcriptional regulation and suggests that MIR630 may be important in controlling the pluripotency characteristics of stem cells. Our findings also suggest that MIR630 may be important in controlling tumorigenesis.

## 4. Materials and Methods

### 4.1. Cell Line and Cell Culture

Human embryonal carcinoma cells (NT2/D1 and NCCIT) were obtained from the Bioresource Collection and Research Center in Taiwan. Cells were incubated in Dulbecco’s Modified Eagle’s Medium (DMEM) (Invitrogen, Carlsbad, CA, USA) supplemented with 10% Fetal bovine serum, 1 mM pyruvate, and 2 mM L-glutamine at 37 °C in a humidified 5% C02/95% air incubator. The undifferentiated cells were split at 1:10 by trypsinization.

### 4.2. MicroRNA Microarray Analysis

NT2/D1 cells were treated with 50 nM PMA for 24 h. Total RNA was purified with Trizol reagent (invitrogen). RNA was quantified using a NanoDrop1000 spectrophotometer (Thermo Fisher Scientific, Inc., Waltham, MA, USA) at OD260nm. RNA integrity was characterized using an RNA 6000 Pico Chip Kit (Agilent Technologies, Santa Clara, CA, USA) on an Agilent 2100 Bioanalyzer. RNA samples with 28S:18S rRNA ratios between 1.4 and 1.8 were used. Briefly, each 100 ng of total RNA was dephosphorylated and labeled with Cy3-pCp using a miRNA Complete Labeling and hybridization Kit (Agilent Technologies). Samples were hybridized using a human miRNA Microarray Kit R16 (Agilent Technologies), which screens for the expression of 1205 human miRNAs and 142 human viral miRNAs. After hybridization, slides were washed with Gene Expression Wash Buffer 1 for 5 min at room temperature, followed by Gene Expression Wash Buffer 2 for 5 min at 37 °C. The slides were scanned on an Agilent G2505C microarray scanner (Agilent Technologies) with default settings. The scanned images were analyzed by GeneSpring 7.3.1 by Welgene Biotech Co., Ltd. (Agilent Technologies). The total gene signal was normalized to the 75th percentile [29]. The normalized signal intensity of each element was compared between untreated and treated cells.

### 4.3. Reverse Transcription-Quantitative Polymerase Chain Reaction (RT-qPCR)

Total RNA was extracted using TRIzol reagent (Invitrogen) according to the protocol recommended by the supplier and reverse-transcribed by Superscript III First-Strand Synthesis System (Invitrogen). RT-qPCR was used to quantify the level of MIR630. Aliquots (0.1 μL) of the cDNA samples were performed in iQ SYBR Green Supermix (Bio-Rad, Hercules, CA, USA) using a Roche LightCycler 480 System. The program consisted of 95 °C for 3 min and 40 cycles of 95 °C for 15 s and 60 °C for 1 min. Data were averaged and relative quantification was determined by the ΔΔCt method. Results were normalized to β2-microglobulin mRNA. Primers used for MIR630 precursor were sense, 5′-CTGAGGTTAAATAACTCCCTC-3′ and antisense, 5′-GTATAGAGCAACCTCTAACAG-3′. The primers used for β2-microglobulin were sense, 5′-GCCGTGTGAACCATGTGACTTT-3′ and antisense, 5′-CCAAATGCGGCATCTTCAAA -3′.

### 4.4. MicroRNA Luciferase Reporter Assay

To contract the luciferase reporters expressing firefly-luciferase in a 3′UTR-dependent manner, pGL3-GAPDH-Luc containing the constitutive GAPDH promoter (16) was modified to replace vector-specific 3′UTR luciferase mRNA with *NANOG*, *POU5F1* (OCT4), or *SOX2* mRNA 3′UTR. To analyze possible microRNA target sites in the 3′ untranslated region (3′UTR) of the *NANOG*, *POU5F1* (OCT4), and *SOX2* genes, the 3′UTR-luciferase reporter vectors were constructed and the effect of miRNA on its activity was evaluated in NT2/D1 cell lines. The 3′UTR fragments of *NANOG*, *POU5F1* (OCT4), and *SOX2* genes were amplified by PCR from genomic DNA of NT2/D1 cells using the following primer pairs containing a XbaI site and a FseI site for the subsequent cloning reactions.

*NANOG* 3′UTR, Forward (XbaI): 5′-cccTCTAGAgaacatgcaacctgaagacgtg-3′ and

Reverse (FseI): 5′-cccGGCCGGCCctgtatatttactcatcgaaac-3′;

*POU5F1* 3′UTR, Forward (XbaI): 5′-cccTCTAGAgctctcccat gcattcaaactg-3′ and

Reverse (FseI): cccGGCCGGCCctgtgtcccaggcttctttat;

*SOX2* 3′UTR, Forward (XbaI): 5′-cccTCTAGAcacactgcccctctcacacatg-3′ and

Reverse (FseI): 5′-cccGGCCGGCCgtgtccatatttcaaaaatttat-3′.

PCR reactions were carried out at 94 °C for 5 min, followed by 35 cycles at 95 °C for 30 s, 50–55 °C for 45 s, and 72 °C for 1 min. PCR products were digested by XbaI and FseI. Desired DNA fragments were PCR purified and inserted into luciferase reporter vector pGL3-GAPDH-Luc via the XbaI and FseI site to generate pGL3-GAPDH-NANOG 3′UTR-Luc, pGL3-GAPDH-POU5F1-3′UTR-Luc, and pGL3-GAPDH-SOX2-3′UTR-Luc. The inserts were positioned in sense orientation relative to the luciferase coding sequence between the XbaI and FseI sites. Proper insertion was verified by direct DNA sequencing.

To analyze possible microRNA target sites in the 3′ untranslated region (3′UTR) of *NANOG*, *POU5F1* (OCT4), and *SOX2* genes, the effect of miRNA mimic on their activity was evaluated in NT2/D1 cell lines. The mirVana miRNA mimics (MIR630 or negative control) were obtained from Ambion (Austin, TX, USA). Transient transfection of the 3′UTR-luciferase reporter vector was performed using Lipofectamine 2000 (Invitrogen) according to the protocol recommended by the supplier. The cells were seeded in 12-well tissue culture plates at 2 × 10^5^ per well 24 h before transfection. On the day of transfection, cells were exposed to DNA–lipofectamine 2000 mixtures containing 0.5 µg of luciferase reporter plasmids, 0.5 µg of pSV-β-galactosidase control vector (Promega, Madison, WI, USA), and 30 nM miRNA mimics. After incubation for 24 h, cells were rinsed with PBS and lysed in 120 µL 1 × reporter lysis buffer (Promega). The lysates were used directly for the Luciferase activity assay (Promega), performed according to the manufacturer’s protocols. The β-Galactosidase enzyme assay (Promega) was performed with the same lysates to standardize transfection efficiencies.

### 4.5. Site-Directed Mutagenesis of the 3′UTR-Luciferase Reporter Vectors

To construct a 3′UTR-luciferase reporter vector lacking the predicted MIR630 binding site, the predicted MIR630 binding sites on *NANOG* were deleted. Site-directed mutagenesis of the 3′UTR luciferase reporter vectors was performed using PCR methods. The PCR reaction mixture contained 1 μg pGL3-GAPDH-Luc containing the 3′UTR of either *NANOG*, *POU5F1*, or *SOX2*, 12.5 µL 2X Phusion High-Fidelity DNA Polymerase (Finnzymes, Espoo, Finland), and 0.1 μg of each primer. The synthetic oligonucleotide primers used were: 5′-TGCCTATCCAGTCAATCTCATGGGTTGGAGCCTAATCAGCGAGGT-3′ (sense) and 5′-ACCTCGCTGATTAGGCTCCAACCCATGAGATTGACTGGATAGGCA-3′ (antisense) for *NANOG* 3′ UTR (124–158), 5′-GAGACGGAGTCTTGCTCTGTCGGTGGCGCGGTCTTGGCTCACTG-3′ (sense) and 5′-CAGTGAGCCAAGACCGCGCCACCGACAGAGCAAGACTCCGTCTC-3′ (antisense) for *NANOG* 3′UTR (254–277), 5′-CTGCTAAGGACAACATTGATAGAATAAGTAGATCTAATACTAGTTTGG-3′(sense) and 5′-CCAAACTAGTATTAGATCTACTTATTCTATCAATGTTGTCCTTAGCAG-3′(antisense) for *NANOG* 3′UTR (570–605), and 5′-AGTTGATTTTACCCTGATTTCTCGATGAGTAAATATACAGGG-3′ (sense) and 5′-CCCTGTATATTTACTCATCGAGAAATCAGGGTAAAATCAACT-3′ (antisense) for *NANOG* 3′UTR (930–952). PCR was performed at 94 °C for 30 s, 55°C for 60 s, and 68 °C for 8 min for 10 cycles. The methylated parental DNA templates were then digested with 20 U DpnI (NEB) at 37 °C for 6 h. The resulting nicked DNA fragments containing the mutation were transformed into competent *E. coli* DH5α cells. The presence of the mutation was verified by direct DNA sequencing.

### 4.6. Preparation of Cell Lysate and Western Blot Analysis

Cells were washed with twice pre-cold PBS and lysed in RIPA Lysis Buffer (Millipore, Bedford, MA, USA). Protein concentrations were quantified by the Bio-Rad protein assay kit (Bio-Rad, Hercules, CA, USA). Protein lysates were resolved on a 10% SDS-polyacrylamide gel and electroblotted onto Immobilon-P PVDF membranes (Millipore). The membranes were blocked in TTBS containing 5% nonfat milk and incubated with diluted primary antibodies overnight at 4 °C. The membranes were then reacted with species-specific HRP-conjugated secondary antibodies (1:5000–10000 in TTBS), and the immunoreactive protein bands were detected by Amersham ECL™ Prime Western Blotting Detection Reagent (GE Healthcare, Chicago, IL, USA). The primary antibodies used included Brachyury monoclonal antibody (1:1000; Millipore), GAPDH monoclonal antibody (1:30000; Chemicon, Temecula, CA, USA), GFAP monoclonal antibody (1:2000; Millipore), NANOG polyclonal antibody (1:2000; Abcam, Cambridge, UK), NeuroD1 polyclonal antibody (1:2500; Millipore), OCT4 monoclonal antibody (1:2000; Cell Signaling Technology, Beverly, MA, USA), Pax6 monoclonal antibody (1:2000; Abnova, Taipei, Taiwan), and SOX2 polyclonal antibody (1:2000; Chemicon).

### 4.7. Statistical Analysis

Data were presented as the mean ± standard deviation of at least three independent experiments, each performed in triplicate. To compare the data between two groups, a two-tailed Student’s t-test was performed. Data from different groups were compared using a one-way ANOVA and a post hoc test with the Holm–Sidak method. The *p* values < 0.05 were considered statistically significant. All analyses were performed using SigmaStat 3.5 (Systat Software, Richmond, CA, USA).

## Figures and Tables

**Table 1:** The top 20 microRNA up-regulated or down-regulated after PMA treatment in NT2/D1 cells.

Upregulated miRNA	Fold of Change	Downregulated miRNA	Fold of Change
hsa-miR-630	28.41	hsa-miR-183	0.44
hsa-miR-335-3p	21.86	hsa-miR-4286	0.47
hsa-miR-335-5p	17.10	hsa-miR-512-3p	0.51
hsa-miR-29b-1*	7.44	hsa-miR-520c-3p	0.54
hsa-miR-365	4.52	hsa-miR-720	0.59
hsa-miR-146a	4.40	hsa-miR-1280	0.59
hsa-miR-1973	4.26	hsa-miR-1260	0.65
hsa-miR-9	4.19	hsa-miR-1274b_v16.0	0.66
hsa-miR-222	4.00	hsa-miR-1260b	0.71
hsa-miR-29b	3.91	hsa-miR-19b-1*	0.72
hsa-miR-4299	3.77	hsa-miR-517a	0.74
hsa-miR-129-3p	3.65	hsa-miR-20b	0.78
hsa-miR-1290	3.64	hsa-miR-367	0.79
hsa-miR-29c	3.50	hsa-miR-551b	0.80
hsa-miR-23a	3.45	hsa-miR-302c*	0.81
hsa-miR-221	3.37	hsa-miR-18b	0.81
hsa-miR-2861	3.22	hsa-miR-19b	0.82
hsa-miR-361-3p	3.05	hsa-miR-20a	0.84
hsa-miR-135b	2.94	hsa-miR-34a	0.85
hsa-miR-423-5p	2.94	hsa-miR-124	0.86

**Table 2:** The predicted microRNA 630 target sites in the 3′UTRs of *NANOG, POU5F1* (encoding for OCT4), and *SOX2*.

Gene	3′UTR Position	Target Site	Minimum Free Energy (MFE)	Target Prediction Program
*NANOG*	124–158	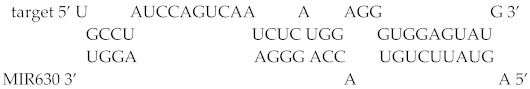	−19.4 kcal/mol	RNAhybrid
*NANOG*	254–277	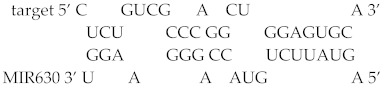	−21.7 kcal/mol	RNAhybrid
*NANOG*	570–605	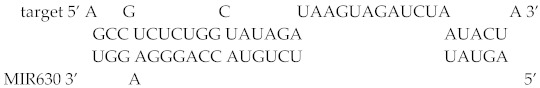	−26.7 kcal/mol	RNAhybrid
*NANOG*	930–952	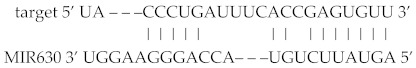	−22.2 kcal/mol (Folding energy)	RNA22
*POU5F1* (OCT4)	9–33	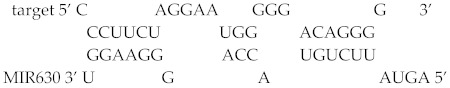	−23.4 kcal/mol	RNAhybrid
*SOX2*	278–301	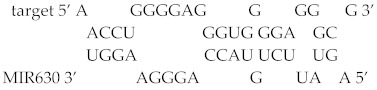	−21.4 kcal/mol	RNAhybrid

## Data Availability

The data that support the findings of this study are available from the corresponding author upon reasonable request.

## Supplementary Materials

The following are available online at https://www.mdpi.com/article/10.3390/ijms23010046/s1.
