# Supplementary material for: MicroRNA 630 Represses NANOG Expression through Transcriptional and Post-Transcriptional Regulation in Human Embryonal Carcinoma Cells
Source: Int J Mol Sci. 2021 Dec 21;23(1):46. doi: 10.3390/ijms23010046 (PMC8744645; doi:10.3390/ijms23010046)
Supplement: Supplementary file 1 [file ijms-23-00046-s001.zip › ijms-1473659-supplementary.pdf]

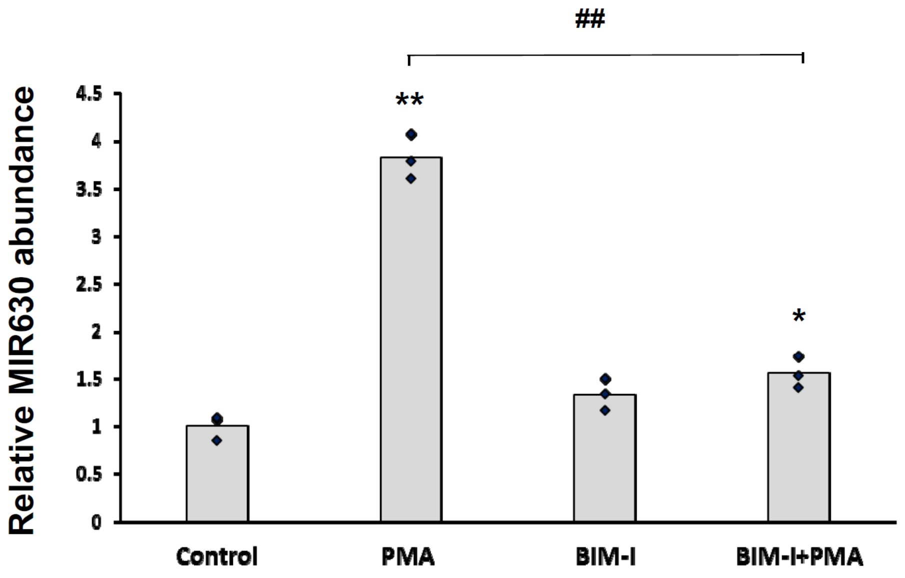

Figure S1. Activation of PKC leads to the induction of MIR630 levels. NT2/D1 cells were treated with 50 nM PMA, pretreated with 25 μM BIM-I for 30 min prior to the addition of PMA, or treated with BIM-I alone for 24 h. MIR630 expression levels were assessed by RT-qPCR, as described in Materials and Methods. Values were normalized with β2-microglobulin mRNA. Significance was tested using one-way analysis of variance (ANOVA) with Holm–Sidak method post hoc test, where * denotes $P<0.05$; ** denotes $P<0.01$ compared with control; where ## denotes $P < 0.01$ compared with cells treated with PMA and “BIM-I+PMA”.”

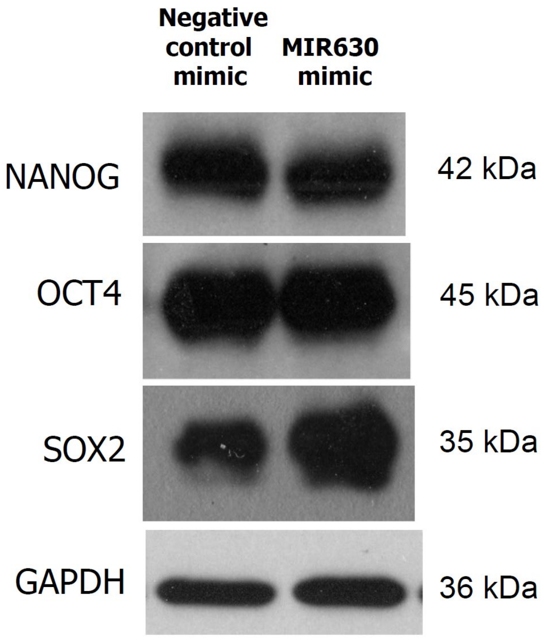

Figure S2. MIR630 mimics did not abolished the overexpression of ectopic NANOG, OCT4 and SOX2. NCCIT cells were cotransfected with pCMV-NANOG, pCMV-OCT4 and pCMV-SOX2 （all at 0.2 μg/ml）along with MI630 mimic or negative control mimic. Cell lysates were prepared 24 h after treatment and analyzed by Western blotting with anti-NANOG, anti-OCT4, anti-SOX2 and anti-GAPDH antibodies.
